# Identification and mapping in spring wheat of genetic factors controlling stem rust resistance and the study of their epistatic interactions across multiple environments

**DOI:** 10.1007/s00122-013-2109-6

**Published:** 2013-05-07

**Authors:** A. Singh, R. E. Knox, R. M. DePauw, A. K. Singh, R. D. Cuthbert, H. L. Campbell, D. Singh, S. Bhavani, T. Fetch, F. Clarke

**Affiliations:** 1 Semiarid Prairie Agricultural Research Center, Agriculture and Agri-Food Canada, Swift Current, SK S9H 3X2 Canada; 2 University of Sydney Plant Breeding Institute Cobbitty, Private Bag 4011, Narellan, 2567 NSW Australia; 3 CIMMYT, Nairobi, Kenya; 4 Cereal Research Center, Agriculture and Agri-Food Canada, Winnipeg, MBT R3T 2M9 Canada

## Abstract

Stem rust (*Puccinia graminis* f. sp. *tritici*) is responsible for major production losses in hexaploid wheat (*Triticum aestivum* L.) around the world. The spread of stem rust race Ug99 and variants is a threat to worldwide wheat production and efforts are ongoing to identify and incorporate resistance. The objectives of this research were to identify quantitative trait loci (QTL) and to study their epistatic interactions for stem rust resistance in a population derived from the Canadian wheat cultivars AC Cadillac and Carberry. A doubled haploid (DH) population was developed and genotyped with DArT^®^ and SSR markers. The parents and DH lines were phenotyped for stem rust severity and infection response to Ug99 and variant races in 2009, 2010 and 2011 in field rust nurseries near Njoro, Kenya, and to North American races in 2011 and 2012 near Swift Current, SK, Canada. Seedling infection type to race TTKSK was assessed in a bio-containment facility in 2009 and 2012 near Morden, MB. Eight QTL for stem rust resistance and three QTL for pseudo-black chaff on nine wheat chromosomes were identified. The phenotypic variance (PV) explained by the stem rust resistance QTL ranged from 2.4 to 48.8 %. AC Cadillac contributed stem rust resistance QTL on chromosomes 2B, 3B, 5B, 6D, 7B and 7D. Carberry contributed resistance QTL on 4B and 5A. Epistatic interactions were observed between loci on 4B and 5B, 4B and 7B, 6D and 3B, 6D and 5B, and 6D and 7B. The stem rust resistance locus on 6D interacted synergistically with 5B to improve the disease resistance through both crossover and non-crossover interactions depending on the environment. Results from this study will assist in planning breeding for stem rust resistance by maximizing QTL main effects and epistatic interactions.

## Introduction

Wheat (*Triticum* *aestivum* L.) is a major cereal crop grown on the prairies of Canada, and yield and quality can be adversely impacted by stem rust. Western Canada is vulnerable to wheat stem rust (*Puccinia* *graminis* Pers. f. sp *tritici*) as exemplified by the race 15B epidemics of 1953 to 1955 which resulted in huge economic losses (Peturson 1958). Globally, stem rust has become a concern with the emergence of Ug99 or TTKS, a race designated after the discovery of virulence to stem rust resistance gene *Sr31* in wheat nurseries in Uganda in 1999 (Pretorius et al. 2000). Recently, two more important stem rust resistance genes *Sr24* (Jin et al. 2008) and *Sr36* (Jin et al. 2009) became ineffective against the TTKS race lineage of stem rust in Kenya. Ug99 and its variants are rapidly evolving making them capable of causing devastating damage to susceptible wheat cultivars in affected regions.

The concern about movement of Ug99 and its variants is justified with the appearance in other parts of the world such as the eastern African highlands, Zimbabwe, Tanzania, South Africa, Sudan, Yemen, and Iran (Singh et al. 2011; Hodson et al. 2012; Pretorius et al. 2012; Hale et al. 2012). The likelihood of long distance dispersal of stem rust was considered high by Watson and de Souza (1983) with their analysis of a past outbreak in which they looked at similarities in characteristics of cultures of stem rust strains from samples taken in Australia and Africa. As part of the North American rust corridor, spread of Ug99 or its variants anywhere into the North American Great Plain would have serious implications for wheat production in Canada. The majority of stem rust spores arriving in Canada derive from rust that overwinters in the south-central part of the USA. The rust is transported through the northward movement of air currents coinciding with progressive northward development of the wheat crop (Fetch et al. 2011). Rust may be spread by wind and water, but the most likely possibility of spread of Ug99 and variants to North America is through human dissemination. Although the majority of the Canadian spring wheat cultivars grown in Canada have moderate to good levels of resistance to the common stem rust races found in North America, the majority of Canadian cultivars are susceptible to Ug99 and its variants.

Growing resistant cultivars is the most efficient, sustainable and environmentally friendly way of controlling rust diseases. Numerous studies have been conducted to understand the genetics of rust resistance. Seedling (typically race specific) and adult plant resistance (APR: typically race non-specific) are two types of rust resistance in wheat characterized based on growth stage. Seedling resistance is effective at all stages of plant growth, whereas APR is expressed as slow rusting and is effective at the post-seedling stage. A single APR gene generally provides partial resistance against a breadth of rust races and alone is not adequate under high disease pressure. To get an adequate level of resistance, gene stacking of three to five APR genes has been recommended (Singh et al. 2000) with the expectation of producing durable resistance over a prolonged period and large area exposed to a favorable disease environment (Johnson 1984). To be pre-emptive in combatting the spread of stem rust, there is a need to identify and incorporate new sources of resistance into Canadian wheat germplasm and release elite cultivars with durable resistance against Ug99 and its variants.

The Canadian cultivar AC Cadillac (DePauw et al. 1998) expresses resistance to Ug99 races TTKST and TTKSK (Hiebert et al. 2011). AC Cadillac has the *SrCad* gene which is a single partially dominant stem rust resistance gene on chromosome 6D linked to the bunt resistance gene *Bt10* (Hiebert et al. 2011). The gene, *SrCad*, alone expresses partial resistance. AC Cadillac may have other unidentified *Sr* genes. Although not proven conclusively, *SrCad* is not considered as another allele of *Sr5* but may be the same as *Sr42* (Hiebert et al. 2011). The genes controlling stem rust resistance in Carberry are unidentified. Both cultivars are considered to have leaf rust resistance gene *Lr34* (linked to or pleiotropic with *Yr18/Sr57/Pm38*), which works synergistically with *SrCad* to give a higher level of resistance (Hiebert et al. 2011). Under some environmental conditions AC Cadillac expresses evidence of pseudo-black chaff (PBC). The *Sr2* gene is linked with the morphological marker PBC, which is characterized by dark pigmentation on glumes or the upper stem (Hare and McIntosh 1979). Out of approximately 50 stem rust resistance genes catalogued, *Sr2* is the only APR gene which has proved durable (McIntosh et al. 1995).

Quantitative trait locus (QTL) mapping is a useful strategy to determine genetic regions controlling stem rust resistance. In addition to genomic regions for *Sr2*/*Yr30* (3B), *Sr57/Lr34*/*Yr18/Pm38* (7D), QTL for APR to stem rust were reported on chromosomes 1A, 2B, 2D, 4A, 4B, 5A, 5B, 6B, and 7A (Bhavani et al. 2011). A QTL mapping study identified a Thatcher APR stem rust resistance QTL on chromosome arm 2BL (Kolmer et al. 2011). In a RIL population derived from HD2009/WL711, QTL for stem rust resistance were identified on chromosomes 3B, 5DL, and 7A (Kaur et al. 2009). In addition, QTL were also identified on chromosomes 1D, 2B, 4B, 5B, and 7D. In a QTL study involving a RIL population derived from Arina/Forno, QTL were mapped on chromosome 5B and 7D along with minor QTL on chromosome 1AS and 7BL (Bansal et al. 2008). Recently, various studies have investigated the relevance of epistatic interactions of genes/QTL for stem rust in durum (Singh et al. 2012), spring wheat (Yu et al. 2011), and winter wheat (Yu et al. 2012). It is important to determine if there is a significant contribution to trait expression from gene interactions in addition to the main effects of QTL. Study of epistatic interaction will provide additional information on the most desirable combinations where genes may function synergistically. For the effective use of genes with quantitative expression in breeding, it will be important also to understand which genes are neutralized or reduce the expression of another gene.

The objective of the present study was to identify and map genomic regions (QTL) associated with stem rust resistance using a DH population derived from a cross of the cultivar Carberry (resistant to North American stem rust races) and AC Cadillac (resistant to North American races, and Ug99 and its variants). We further investigated the epistatic interactions between identified QTL to assist in the understanding of beneficial combinations for stem rust resistance in multiple environments.

## Materials and methods

### Plant materials

A DH population was developed at the Semiarid Prairie Agricultural Research Centre from Carberry/AC Cadillac using the maize pollen method described by Knox et al. (2000). Carberry is a doubled haploid hexaploid wheat cultivar resistant to the prevalent Canadian prairie races of stem rust (DePauw et al. 2011). AC Cadillac is a hexaploid wheat cultivar (DePauw et al. 1998) resistant to Canadian prairie stem rust races as well as Ug99 and its variant races (Hiebert et al. 2011). Two hundred and sixty-one DH lines selected from a winter increase nursery near Leeston, New Zealand in 2009 were used in this study. Sixty-three lines prior to being sent to Leeston had undergone selection in a disease nursery near Swift Current, Saskatchewan, Canada in 2008 to remove tall, late maturing, stem rust and common bunt susceptible lines.

### Stem rust assessment

Stem rust seedling inoculation of the 261 lines and parents during 2009 and 2012 was performed in the biological containment facility near Morden, Manitoba, Canada using the method described by Hiebert et al. (2011). Seedlings were inoculated with stem rust race TTKSK. Infection-type ratings were completed approximately 2 weeks after inoculation using the scale described by Stakman et al. (1962).

Parents and the 261 lines were grown in an unreplicated test at the Kenyan Agricultural Research Institute, Njoro, Kenya for response to stem rust in nurseries in 2009, 2010 and 2011. Parents were replicated in each nursery. About 2 g of seed per entry was planted in twin 1 m rows spaced 30 cm apart. Suspension of urediniospores of both *Sr24* and *Sr31* virulent races in lightweight mineral oil Soltrol 170 was misted on to the spreader rows at jointing to initiate disease development. Stem rust severity was rated as 0 to 100 % and was recorded using a modified Cobb scale (Peterson et al. 1948). The stem rust infection response was recorded as R, R-MR, MR, M, MR-MS, MS, MS-S, S, where R is resistant, S is susceptible and M is moderate. The infection response lying between any two categories is denoted by a hyphen. Ratings were recorded up to two times between heading and plant maturity. Pseudo-black chaff symptoms were reported as present or absent in Kenya (2010 and 2011) and in Canada (2012) using a two point scale: 1 = PBC present, and 2 = PBC absent. Stem rust and PBC ratings were recorded simultaneously.

The parents and the 261 DH lines were also evaluated for reaction to stem rust in a field nursery near Swift Current, Saskatchewan, Canada in 2011 and 2012 where rust susceptible spreader rows were needle inoculated using a mixture of races representative of virulence in North America [see DePauw et al. (2011) for races used]. Stem rust severity and infection response were rated as described above at the early dough stage.

For quantitative analysis, the Stakman scale was converted to a numeric scale such that symbol ‘0’ = 0, ‘;’ = 1, ‘1−’ = 2, ‘1’ = 3, ‘1+’ = 4, ‘2−’ = 5 and so on. Similarly, Field Response classes were converted to numeric values. Homogeneity of variance testing was performed to determine the appropriateness of combining metric data over environments. Bartlett’s χ^2^ test was calculated in SAS (SAS Institute, 2009) to test for homogeneity of variances prior to combining data in the following nurseries: stem rust infection type (indoor screening at Morden) (2009 and 2012), Kenya severity (2009, 2010, 2011), Kenya infection response (2009, 2010, 2011), Swift Current severity (2011, 2012) Swift Current infection response (2011, 2012), and PBC (2010, 2011).

### Molecular genotyping

The DNA was extracted from the parents and 261 DH lines for PCR using the Wheat and Barley DNA Extraction in 96-well Plates protocol (http://maswheat.ucdavis.edu/PDF/DNA0003.pdf) with modifications as described hence. When the plants reached the 1 to 2 leaf stage, 3 cm leaf segments from primary leaves were harvested for genomic DNA isolation. A 10 μl PCR reaction consisting of DNA (final concentration of 2.4 ng/μl), Ultrapure double distilled H_2_0 (Gibco), 10 % PCR Buffer without MgCl_2_ [Invitrogen cat.#18067-017: 200 mM Tris–HCl (pH 8.4), 500 mM KCl], 10 mM dNTPs (Roche), 1.5 mM MgCl_2_ (Invitrogen), 0.07 U μl^−1^ Taq (5 U of activity μl^−1^) NEB, and 2 ηg μl^−1^ forward and 2 ηg μl^−1^ reverse primer was used for the DNA amplification process. The PCR conditions were: initial denaturation at 94 °C (3 min), followed by 44 cycles of 94 °C (1 min), 55 or 60 °C annealing (1 min), and 72 °C extension (1 min) with a final extension at 72 °C for 10 min. The amplification products were resolved by capillary electrophoresis using an ABI3130XL DNA fragment analyser (Applied Biosystems), or by horizontal high resolution agarose electrophoresis using 2 % Metaphor and 1 % agarose LE gels at 4 V cm^−1^ in TBE (0.045 M TRIS, 0.045 M Borate, and 0.001 M EDTA) buffer and stained with ethidium bromide (0.5 μg ml^−1^). The DNA banding patterns were visualized with UV light and recorded by a Kodak, Gel Logic 100 digital camera imaging system. The size of bands run on agarose was determined by comparing against a 50 bp DNA ladder (Fermentas).

Fifty-eight simple sequence repeat (SSR) and 578 Diversity Array Technology (DArT®)  polymorphic markers were used to genotype the 261 lines along with the parents. DArT® marker screening was done by Triticarte Pty. Ltd. Yarralumla, ACT, Australia (www.triticarte.com.au). The DNA was extracted from parents and DH lines for DArT^®^ analysis according to protocol published by Triticarte (http://www.triticarte.com.au/pdf/DArT_DNA_isolation.pdf).

### QTL analysis

A genetic linkage map was constructed with software JoinMap^®^ 4.0 using the regression mapping option and groupings were created using independence LOD (Van Ooijen 2006). Centimorgan (cM) values were calculated according to the Haldane mapping function. Each linkage group was assigned to the corresponding hexaploid wheat chromosome based on the known genomic positions of the DArT^®^ and SSR markers in the groups. The QTL mapping was performed using MapQTL6^®^ (Van Ooijen 2009) to identify molecular markers significantly associated with stem rust resistance. Logarithm of the Odds (LOD) threshold for significance was obtained by MapQTL^®^’s permutation test option (1,000 permutations). Genome-wide threshold levels were used to declare significant QTL based at a 5 % significance level. Automatic co-factor detection based on backward elimination as well as manual co-factor selection was used to identify the co-factor markers for Multiple QTL Mapping (MQM).

### Epistasis analysis

The interactions of QTL were identified using the software QTLNetwork version 2.1 (Yang et al. 2008). This software can map both single-locus effect QTL and epistasis. QTL effects were estimated by the mixed linear model (MLM) approach. A “2D genome scan” option was used to map epistatic QTL with or without single-locus effects. Using the option “map epistasis”, epistatic effects of additive × additive (A*A) were mapped because Carberry/AC Cadillac is a DH population. Using the “permutation” option, critical F values were calculated to control the experimental type I error rate by the permutation test.

## Results

### Stem rust reaction

**Fig. 1:**
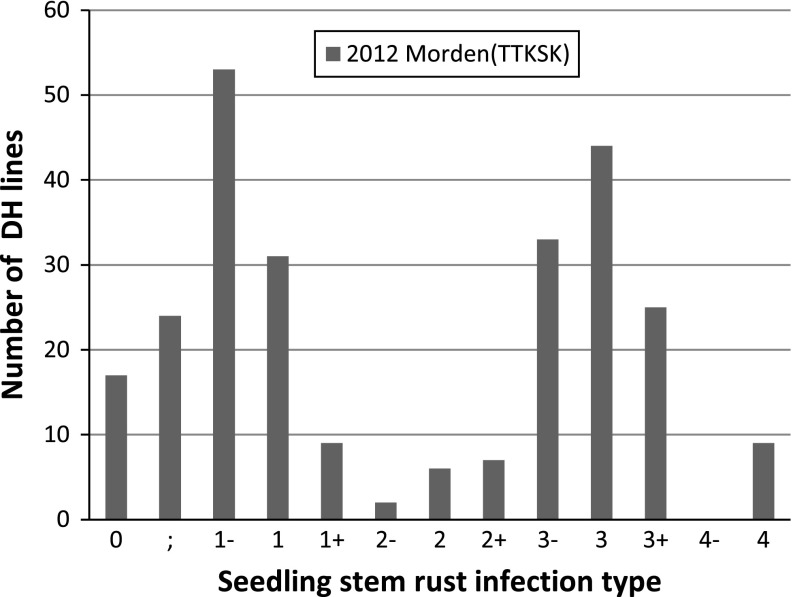
Frequency distribution of seedling stem rust infection type of the Carberry/AC Cadillac doubled haploid (DH) population measured in the bio-containment facility at Morden to TTKSK in 2012. In 2012, AC Cadillac showed a mean rating of 1 and Carberry showed a mean rating of 3 to 3+

Stem rust seedling infection-type data from Morden 2009 and 2012 were bimodally distributed. Figure 1 shows the frequency distribution for Morden 2012. The Morden 2009 results were similarly distributed. Stem rust developed less on AC Cadillac than Carberry. In 2012, the mean rating of AC Cadillac for seedling infection type was 1 while Carberry was between 3 and 3 + . Lines expressing a lower seedling infection type than the resistant parent (AC Cadillac) as well as lines exhibiting a higher seedling infection type than Carberry were observed in both trials. For example in Fig. 1, lines rated zero were observed at the resistant end of the distribution and lines rated four were observed at the susceptible end of the distribution.

**Fig. 2:**
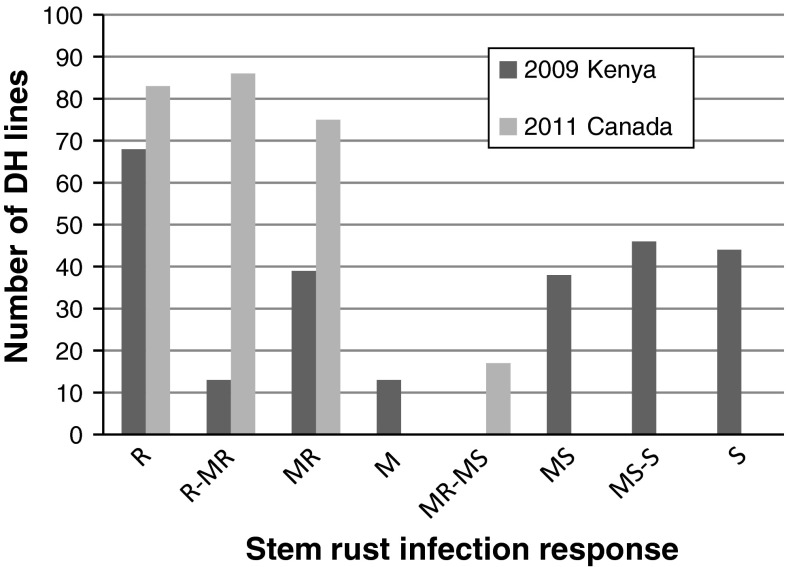
Frequency distribution of stem rust infection response of the Carberry/AC Cadillac doubled haploid (DH) population. Measurements of Ug99 stem rust reaction made in field nurseries near Njoro, Kenya in 2009 and of North American races near Swift Current, Canada in 2011 are presented. In Kenya, AC Cadillac infection response varied from R-MR to M and Carberry expressed infection response from M to MS-S whereas in Canada, AC Cadillac showed an MR infection response while Carberry showed R-MR

In Kenya, across years the AC Cadillac infection response varied from R-MR to M and Carberry showed an infection response from M to MS-S; whereas in Canada AC Cadillac showed an MR infection response while Carberry rated R-MR. The infection response of the population varied from R to S in 2009 and 2011 in Kenya field tests and from R to MS-S in 2010. The infection response of the population to stem rust in Canada in 2011 varied from R to MR-MS and R to M in 2012. Population lines with a more susceptible response than AC Cadillac and Carberry were observed in both Kenya and Canada. In all environments except Kenya 2009, a preponderance of lines was observed in the more resistant classes. Figure 2 shows the example of Canada 2011 where stem rust infection response is distributed more into the resistant classes and Kenya 2009 where infection response is distributed more evenly across resistant and susceptible classes.

**Fig. 3:**
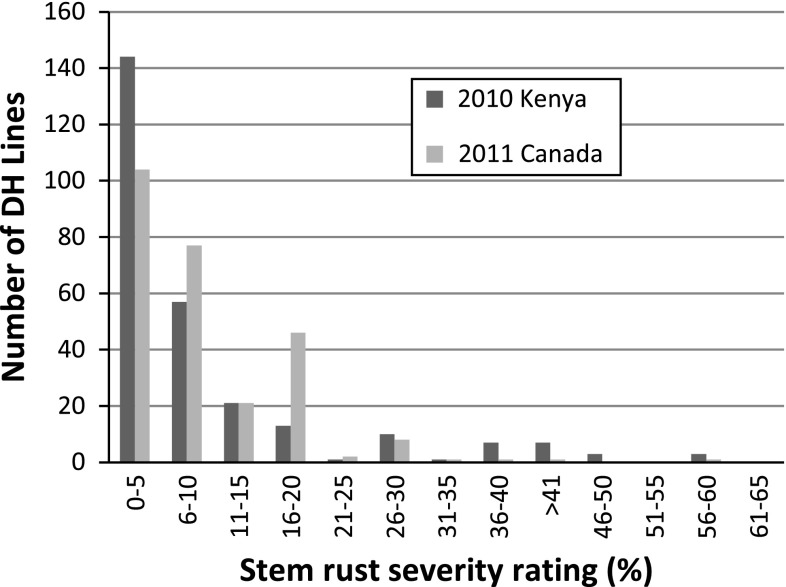
Frequency distribution of stem rust severity (%) ratings of the Carberry/AC Cadillac doubled haploid (DH) population. Measurements of Ug99 stem rust severity made in field nurseries near Njoro, Kenya in 2010 and of North American races in Swift Current, Canada 2011 presented. In Njoro, Kenya, the mean disease severity of AC Cadillac was 1.7 % in 2010, while the mean disease severity of Carberry was 17.5 % in 2010. The mean disease severity of AC Cadillac near Swift Current, Canada was 11.7 % in 2011 and Carberry mean severity was 9.2 % in 2011

Homogeneity tests indicated the data were homogeneous for Stem rust infection type (Bartlett’s Chi square test *P* value = 0.92), Kenya infection response (*P* value = 0.55), Canada severity (*P* value = 0.9979), Canada infection response (*P* value = 1) and PBC (*P* value = 1), while data were not homogenous for Kenya severity (*P* value < 0.0001). Genotypes were a significant source of variation for stem rust infection type (*P* value < 0.0001), Kenya severity (*P* value < 0.0001), Kenya infection response (*P* value < 0.0001), Canada severity (*P* value < 0.0001), Canada infection response (*P* value < 0.0001), but not for PBC (*P* value = 0.93). In Njoro, Kenya, the mean disease severity of AC Cadillac was 3.4 % in 2009, 1.7 % in 2010 and 6.7 % in 2011, while the mean disease severity of Carberry was 18.3 % in 2009, 17.5 % in 2010 and 13.3 % in 2011. The disease severity of the DH lines varied from zero or little up to 50 % in 2009, 60 % in 2010, and 70 % in 2011. The mean disease severity of AC Cadillac in Canada was 11.7 % in 2011 and 5.9 % in 2012, and Carberry mean severity was 9.2 % in 2011 and 2.9 % in 2012, while the disease severity in the population was as high as 60 % in 2011 and 20 % in 2012. Several DH lines were noted with lower severity than AC Cadillac in all environments. Doubled haploid lines with higher stem rust severity than both parents were also observed. As with infection response, the population was skewed for severity in all environments with a preponderance of lines in the resistant end of the distribution as demonstrated in Fig. 3 for Kenya 2010 and Canada 2011 severity.

### QTL mapping

Bartlett’s Chi square homogeneity test on converted class data indicated homogeneity for Morden converted infection type (*P* value = 0.92), Kenya converted infection response (*P* value = 0.55), and Canada converted infection response (*P* value = 1). Therefore, QTL analysis was performed on data combined over environments where variances were homogeneous.

**Fig. 4:**
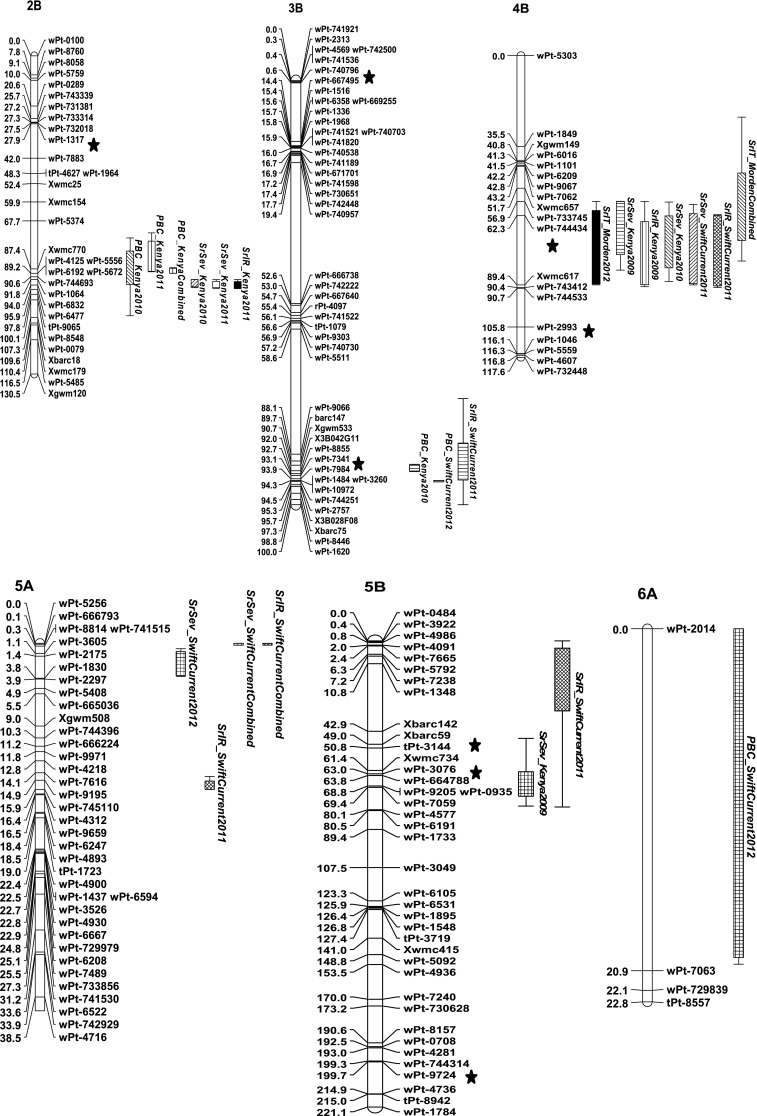

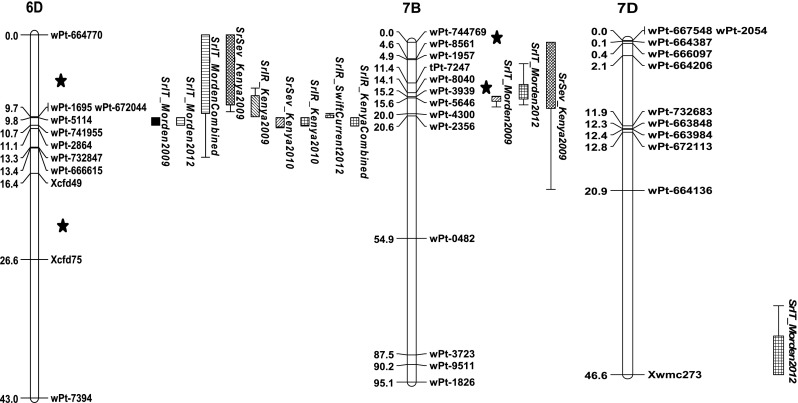
Stem rust resistance QTL identified on chromosome 2B, 3B, 4B, 5, 5B, 6, 7B and 7D and pseudo-black chaff (PBC) QTL identified on chromosome 2B, 3B and 6A, using DArT and SSR markers in a doubled haploid population derived from Carberry/AC Cadillac. Disease reactions for stem rust severity, infection response and PBC were assessed near Njoro, Kenya (2009, 2010 and 2011) and near Swift Current, Canada (2011 and 2012), and seedling stem rust infection type was assessed in Morden, Canada (2009 and 2012). Location of a QTL involved in epistasis is depicted with the symbol *asterisk*

Eight QTL for stem rust resistance and three QTL for PBC on nine wheat chromosomes were identified (Table 1; Fig. 4). Table 1 presents significant QTL, the mean of each measurement variable for each marker molecular variant as they relate to the parents, and the proportion of variation explained by the QTL. In some cases QTL (main effect and epistatic effect QTL that will be discussed later) appeared on the same chromosome, but it was unclear whether they mapped to different regions (Fig. 4). These QTL were assigned distinguishing number symbols (Table 1).

**Table 1:** Parental-type molecular variant mean values for each marker, most significant marker or marker interval, LOD, measure of additive effects and percent regression effect explained from Multiple QTL Mapping using MapQTL in the Carberry/AC Cadillac doubled haploid population for DArT^®^ and SSR markers with stem rust severity and infection response from the field in Njoro, Kenya and Swift Current, Canada, and for seedling stem rust infection type against race TTKSK from containment growth chamber trials in Morden, Canada

Chromosome	QTL	Trait	Environment	Marker^a^/Marker interval	LOD^b^ score	Mean AC Cadillac molecular variant	Mean Carberry molecular variant	Additive^c^ effect	PV^d^ %
2B	*QSr.spa*-*2B.1*	Stem rust severity	Kenya 2010	*wPt*-*6832*	7.4	6.4	14	−3.8	10.3
	*QSr.spa*-*2B.1*	Stem rust severity	Kenya 2011	*tPt*-*9065*	7	10.3	12.6	−1.1	9.7
	*QSr.spa*-*2B.1*	Stem rust infection response	Kenya 2011	*wPt*-*6832*	3.7	1.7	3.4	−0.8	6.3
	*QPbc.spa*-*2B*	PBC	Kenya 2010	*Xwmc770*	3.2	1.7	1.5	0.1	4.9
	*QPbc.spa*-*2B*	PBC	Kenya 2011	*Xwmc770*	7.1	1.3	1.1	0.1	11.7
3B	*QSr.spa*-*3B.1*	Stem rust infection response	Canada 2011	*Xbarc147*	3	1.9	2.4	−0.2	4.6
	*QPbc.spa*-*3B*	PBC	Kenya 2010	*X3B042G11*	8.1	1.8	1.4	0.2	13.7
	*QPbc.spa*-*3B*	PBC	Canada 2012	*wPt*-*744251*	12	1.3	1	0.2	18
4B	*QSr.spa*-*4B.1*	Stem rust infection type	Morden 2012	*wPt*-*744434*–*Xwmc617*	3.1	5.9	4.2	0.8	2.8
	*QSr.spa*-*4B.1*	Stem rust infection response	Kenya 2009	*wPt*-*744434*–*Xwmc617*	2.9	4.8	3.7	0.5	2.4
	*QSr.spa*-*4B.1*	Stem rust severity	Kenya 2009	*wPt*-*744434*	4	12.3	7.9	2.2	5.8
	*QSr.spa*-*4B.1*	Stem rust severity	Kenya 2010	*wPt*-*744434*	4.5	13.4	7	3.2	6.1
	*QSr.spa*-*4B.1*	Stem rust severity	Canada 2011	*wPt*-*744434*–*Xwmc617*	3.4	14.3	9	2.6	5.3
	*QSr.spa*-*4B.1*	Stem rust infection response	Canada 2011	*wPt*-*744434*–*Xwmc617*	3	2.6	2	0.3	4.7
	*QSr.spa*-*4B.1*	Stem rust infection type	Morden combined	*wPt*-*733745*	3	6.3	4.8	0.8	5.1
5A	*QSr.spa*-*5A*	Stem rust infection response	Canada 2011	*wPt*-*5408*	2.9	2.5	2	0.2	4.5
	*QSr.spa*-*5A*	Stem rust severity	Canada 2012	*wPt*-*2175*	3.2	3.7	2.1	0.8	5.5
	*QSr.spa*-*5A*	Stem rust severity	Canada Combined	wPt-666793	3.1	8.2	6	1.1	5.3
	*QSr.spa*-*5A*	Stem rust infection response	Canada combined	wPt-5256	3.4	2	1.6	0.2	5.9
5B	*QSr.spa*-*5B.1*	Stem rust severity	Kenya 2009	*wPt*-*9205*	3.6	8.2	12	−1.9	4.9
	*QSr.spa*-*5B.1*	Stem rust infection response	Canada 2011	*wPt*-*5792*	3.6	2	2.5	−0.3	5.4
6A	*QPbc.spa*-*6A*	PBC	Canada 2012	*wPt*-*2014*–*tPt*-*8557*	3.2	1.1	1.3	−0.1	4.4
6D	*QSr.spa*-*6D*	Stem rust infection type	Morden 2012	*wPt*-*741955*	34.7	2.5	7.5	−2.5	42.8
	*QSr.spa*-*6D*	Stem rust infection type	Morden 2009	*wPt*-*1695*	39.2	2.7	7.5	−2.4	48.8
	*QSr.spa*-*6D*	Stem rust infection response	Kenya 2009	*wPt*-*1695*	39.8	2.3	6.1	−1.9	47.8
	*QSr.spa*-*6D*	Stem rust severity	Kenya 2009	*wPt*-*664770*	8.5	7	13.2	−3.1	12.8
	*QSr.spa*-*6D*	Stem rust severity	Kenya 2010	*wPt*-*741955*	2.9	7.8	12.6	−2.4	3.9
	*QSr.spa*-*6D*	Stem rust infection response	Kenya 2010	*wPt*-*741955*	13.4	1.6	3.4	−0.9	21.1
	*QSr.spa*-*6D*	Stem rust infection response	Canada 2012	*wPt*-*1695*	3.4	1.1	1.4	−0.2	5.8
	*QSr.spa*-*6D*	Stem rust infection type	Morden combined	*wPt*-*741955*	9.4	3.8	6.4	−1.3	15
	*QSr.spa*-*6D*	Stem rust infection response	Kenya combined	*wPt*-*741955*	6.7	2.6	3.7	−0.5	11.1
7B	*QSr.spa*-*7B*	Stem rust infection type	Morden 2012	*wPt*-*3939*	8.6	3.8	5.8	−1	7.1
	*QSr.spa*-*7B*	Stem rust infection type	Morden 2009	*wPt*-*3939*	3.6	3.4	6.8	−1.7	3.2
	*QSr.spa*-*7B*	Stem rust severity	Kenya 2009	*wPt*-*3939*	3.2	8.1	11.6	−1.8	4.3
7D	*QSr.spa*-*7D*	Stem rust infection type	Morden 2012	*Xwmc273*	4.4	4.4	6	−0.8	3.8

^a^Marker interval described by the markers which immediately flank the peak QTL response, or in the case of a single marker, the marker which is at the peak QTL response

^b^The threshold to declare LOD scores significantly ranged from 2.9 to 3.0. All LOD scores reported are significant

^c^A positive additive effect indicates Carberry contributed to stem rust resistance and a negative additive effect indicates AC Cadillac contributed to stem rust resistance

^d^PV is the proportion of the phenotypic variance explained by the QTL

The main effect QTL that appeared only for traits measured in Kenya or to TTKSK tested at Morden were *QSr.spa*-*2B.1*, *QPbc.spa*-*2B*, *QSr.spa*-*5B.1*, *QSr.spa*-*7B*, and *QSr.spa*-*7D* (Table 1). Those QTL that appeared only for traits measured in Canada were *QSr.spa*-*3B.1*, *QSr.spa*-*5A*, *QSr.spa*-*5B.1*, and *QPbc.spa*-*6A* and those QTL that appeared for traits measured in Kenya and Canada were *QPbc.spa*-*3B.1*, *QSr.spa*-*4B.1*, and *QSr.spa*-*6D*.

The significant QTL LOD scores ranged from 2.9 to a high of 39.8 (Table 1). The highest LOD scores were associated with *QSr.spa*-*6D* which was effective against stem rust in the greatest number of environments and for all measures of rust resistance. Other relatively high LOD scores for rust resistance were associated with *QSr.spa*-*7B* and *QSr.spa*-*2B.1.* The QTL with the highest LOD scores were also the loci that explained the greatest amount of the phenotypic variation associated with the trait. Although *QSr.spa*-*4B.1* did not explain much of the PV, it appeared across multiple environments and rust resistance measures.

AC Cadillac contributed resistance at the *QSr.spa*-*2B.1, QSr.spa*-*3B.1, QSr.spa*-*5B.1, QSr.spa*-*6D, QSr.spa*-*7B* and *QSr.spa*-*7D* loci while Carberry contributed resistance at the *QSr.spa*-*4B.1* and *QSr.spa*-*5A* (Table 1). The association of resistance with a particular parent at a particular locus was consistent across rust resistance measures and environments. For example, the rust resistance at *QSr.spa*-*4B.1* was contributed by Carberry in both Kenya and Canada, for adult and seedling reactions, for severity and infection response and for multiple years. Pseudo-black chaff was contributed by AC Cadillac at *QPbc.spa*-*2B*, and *QPbc.spa*-*3B* and by Carberry at *QPbc.spa*-*6A*. Except for *QPbc.spa*-*6A*, PBC QTL coincided with stem rust resistance QTL.

The QTL for seedling resistance, *QSr.spa*-*7B, QSr.spa*-*7D*, *QSr.spa*-*6D*, and *QSr.spa*-*4B.1,* did not always translate into QTL for adult plant resistance (Fig. 4). Those QTL that were effective in both seedling and adult stages were *QSr.spa*-*6D,* and *QSr.spa*-*4B.* Infection response and severity QTL occurred at the same locus in half the occurrences of resistance QTL, although usually not in the same environment (Fig. 4).

### Epistasis analysis

Significant epistatic interactions were identified for stem rust severity, infection response and seedling infection type (Table 2). The largest epistatic interaction was for stem rust severity between *QSr.spa*-*5B.1* and *QSr.spa*-*6D* with the estimated additive by additive interaction effect (A*A) of 0.78**, while the next largest epistatic interaction, also for severity, was between *QSr.spa*-*4B.1* and *QSr.spa*-*7B*. Significant epistatic interactions were also detected for stem rust infection response between *QSr.spa*-*5B.1* and *QSr.spa*-*6D* (A*A = 0.12**), *QSr.spa*-*3B.1* and *QSr.spa*-*6D* (A*A = 0.18***) and between *QSr.spa*-*4B.2* and *QSr.spa*-*5B.2* (A*A = −0.19***). Epistatic interaction for stem rust seedling infection type between *QSr.spa*-*6D* and *QSr.spa*-*7B* was also observed (A*A = 0.32**).

**Table 2:** Estimated additive × additive epistatic (A*A) effects of QTL detected by two-locus interaction analysis using QTL. Network for stem rust severity and infection response in the field in Kenya (2009, 2010, 2011), Canada (2011, 2012) and for seedling stem rust infection type against race TTKSK in growth chamber trials in Morden (2009 and 2012), Canada in the doubled haploid population derived from Carberry/AC Cadillac

Trait	QTL_1_^a^	Flanking interval_1_^a^	QTL_2_^b^	Flanking interval_2_^b^	A_1_*A_2 effect_^c^
Stem rust severity	*QSr.spa*-*4B.1*	*wPt*-*744434*–*Xwmc*-*617*	*QSr.spa*-*7B*	*wPt*-*744769*–*wPt*-*8561*	−0.59*
	*QSr.spa*-*6D*	*wPt*-*664770*–*wPt*-*1695*	*QSr.spa*-*5B.1*	*tPt*-*3144*–*Xwmc734*	0.78**
Stem rust infection response	*QSr.spa*-*6D*	*wPt*-*664770*–*wPt*-*1695*	*QSr.spa*-*5B.1*	*wPt*-*664788*–*wPt*-*9205*	0.12**
	*QSr.spa*-*3B.1*	*wPt*-*7341*–*wPt*-*7984*	QSr*.spa*-*6D*	*Xcfd49*–*Xcfd75*	0.18***
	*QSr.spa*-*4B.2*	*wPt*-*2993*–*wPt*-*1046*	*QSr.spa*-*5B.2*	*wPt*-*9724*–*wPt*-*4736*	−0.19***
Stem rust seedling infection type	*QSr.spa*-*6D*	*wPt*-*664770*–*wPt*-*1695*	*QSr.spa*-*7B*	*wPt*-*3939*–*wPt*-*5646*	0.32**

^a^First QTL_1_ and interval of a pair of interacting QTL

^b^Second QTL_2_ and interval of a pair of interacting QTL

^c^A_1_*A_2_ is the additive x additive interaction or epistatic effect across environments

Probability levels: * significant at 5 %; ** significant at 1 %, and *** significant at 0.1 %

**Fig. 5:**
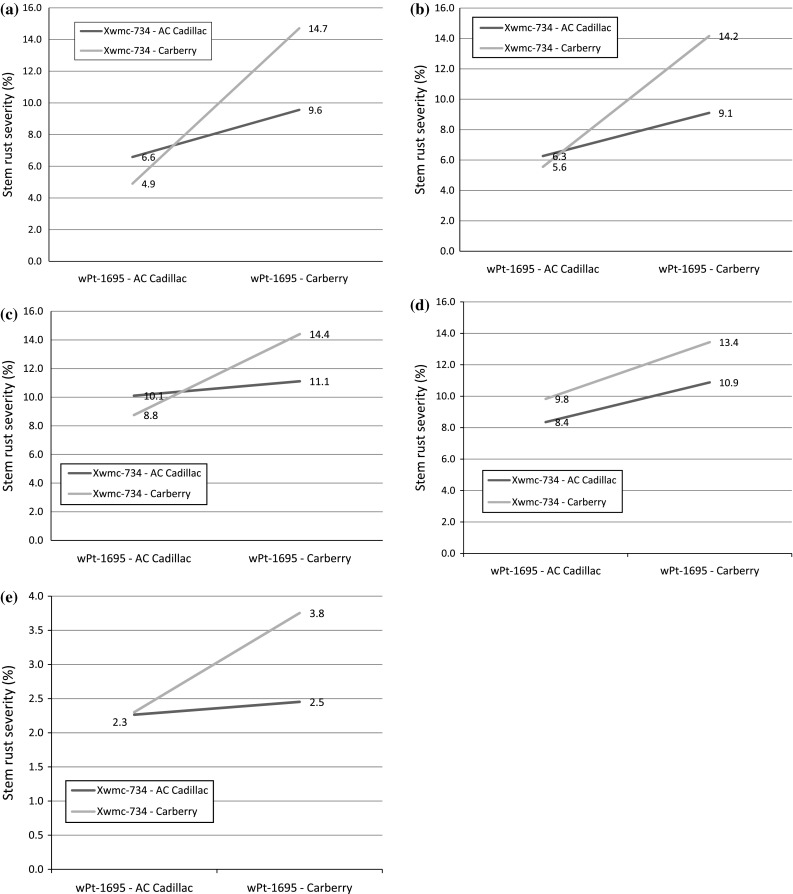
Interaction of stem rust severity (%) between DArT marker *wPt-1695* on chromosome 6D and SSR marker *Xwmc-734* on chromosome 5B: **a** Njoro, Kenya (2009), **b** Njoro, Kenya (2010), **c** Njoro, Kenya (2011), **d** Swift Current, Canada (2011), **e** Swift Current, Canada (2012)

In order to decipher the genetic architecture of the largest A*A effect, we looked at the molecular variants of the significant interacting loci *QSr.spa*-*5B.2* and *QSr.spa*-*6D* for stem rust severity (Table 2). A crossover interaction was detected for stem rust severity near markers *wPt*-*1695* and *Xwmc734*. Since the results were similar when we looked at the other flanking markers (*wPt*-*664770* or *wPt*-*1695* with *tPt*-*3144* or *Xwmc734)* we only present the interaction of *wPT*-*1695* (fewer missing data points than *wPt*-*664770*) and *Xwmc734* (SSR locus and can be easily anchored to available maps). The crossover interaction between *wPt*-*1695* and *Xwmc734* at Kenya in 2009, 2010 and 2011 can be visualized in Fig. 5a–c and non-crossover interactions for 2010 and 2011 in Canada are shown in Fig. 5d–e. The mean stem rust severity was numerically lowest in Kenya with the combination of *wPt*-*1695*-AC Cadillac molecular variant and *Xwmc734*-Carberry molecular variant in all three Kenya environments.

## Discussion

The QTL derived from AC Cadillac with a major effect on resistance at the seedling stage identified on chromosome 6D, *QSr.spa*-*6D*, from the Carberry/AC Cadillac DH population was consistent with the phenotypic distribution. That is to say the bimodal distribution with similarly sized peaks also indicated the segregation of a major seedling resistance gene to race TTKSK. The wide range of reaction within each peak indicated the presence of modifier genes and the QTL analysis indicated small effect or modifier genes at other loci for seedling stage infection-type resistance on chromosomes 4B, 7B, and 7D.

Expression of the *QSr.spa*-*6D* conditioned seedling resistance was carried through to the adult plant stage with field ratings also revealing the QTL on 6D. The influence of additional genetic factors was suggested by the positive skew of frequency distributions for field stem rust severity and infection response in which the majority of lines showed low disease severity and infection. The hypothesis of multiple gene segregation within the Carberry/AC Cadillac population was borne out by the identification of several QTL on other chromosomes.

*QSr.spa*-*6D* appears to be a broad and consistently expressed source of resistance, being significant in both Kenyan and Canadian environments in multiple years. Based on the marker and map position, *QSr.spa*-*6D* likely encompasses the seedling resistance gene *SrCad* (Hiebert 2011). Based on marker analysis, pedigree, leaf tip necrosis, phenotype, and lack of segregation within breeding populations, both AC Cadillac and Carberry are believed to possess the *Lr34* gene. The level of stem rust resistance conditioned by *SrCad* is enhanced by the presence of *Lr34* compared to *SrCad* alone (Hiebert et al. 2011). The greater number of resistant lines in the Carberry/AC Cadillac population identified by Kenya field ratings compared to the segregation pattern of seedlings indicated more genes expressed in adult plants. The influence of APR is further supported by the multiple QTL uniquely associated with adult plant field ratings. Our results extend the knowledge surrounding the *QSr.spa*-*6D* QTL by revealing the epistatic interaction with other loci. For example, the QTL on 6D interacted with *QSr.spa*-*5B* on 5B for stem rust infection response and severity, and with *QSr.spa*-*7B* on 7B for seedling infection type. While non-crossover interactions were observed for results from the Canadian nurseries, a crossover interaction was observed in all three Kenya nurseries suggesting transgressive segregation could be achieved by recombining the allele in *QSr.spa*-*6D* from AC Cadillac with the allele in *QSr.spa*-*5B.1* from Carberry. This information can assist breeders to utilize the epistatic interactions for the improvement of rust resistance. In this case, the combination of the AC Cadillac molecular variant on 6D along with the Carberry molecular variant on 5B should be utilized in marker assisted selection.

The level of significance of *QSr.spa*-*2B.1* and its appearance in 2 years of Kenyan nursery testing suggested this QTL has merit for control of Kenyan races of stem rust. Although the level of PV explained for rust severity is modest, this locus would be worth targeting in crosses where Carberry was planned as a parent. *QSr.spa*-*2B.1* may be the same gene previously reported associated with stem rust and yellow rust resistance in an association mapping study of historical wheat conducted by Crossa et al. (2007). The 2BS region has been associated with stem rust resistance genes such as *Sr19, Sr23, Sr36* and *Sr40* (Crossa et al. 2007).

On chromosome 2B, the stem rust QTL, *QSr.spa*-*2B.1* mapped in the same region as the PBC QTL, *QPbc.spa*-*2B.* A PBC QTL on chromosome 2B was also reported by Kaur et al. (2009). These results suggest that PBC can be associated with a stem rust resistance gene other than for *Sr2*. That being said, our results indicated weak expression for PBC and stem rust resistance in the *Sr2* region with the peak of the *QSr.spa*-*3B* QTL mapped at SSR marker *Xbarc147,* 1 cM from *Xgwm533* which McNeil et al. (2008) demonstrated is linked to *Sr2*. Consistent with the demonstrated linkage of *Sr2* with a PBC locus which was demonstrated by McNeil et al. (2008), we observed in the Carberry/AC Cadillac population that PBC was influenced by the QTL *QPbc.spa*-*3B* on chromosome 3B. The *QPbc.spa*-*3B* mapped to the same genomic region as PBC QTL *QPbc.sun*-*3B* reported by Kaur et al. (2009). The proportion of rust resistance PV explained by *QSr.spa*-*3B.1* (Table 1) was much lower than the PV (11.6–32.2 %) explained by the *QSr.cim*-*3B* QTL reported by Njau et al. (2012), possibly indicating more than one allele for *Sr2*. The appearance of the PBC QTL *QPbc.spa*-*6A* in Canada 2012, albeit showing a minor effect, demonstrates the complexity of genetic control of the PBC trait. *QPbc.spa*-*6A* was mapped in the same genomic region on chromosome arm 6AS as reported by Bariana et al. (2001). This PBC QTL appeared in a single environment but unlike the other PBC QTL, no association with stem rust resistance was observed.

The *QSr.spa*-*4B.1* QTL from Carberry was notable for its consistency, appearing at seedling and adult stages, over more than one year under field conditions and in both Kenya and Canada. Unfortunately this QTL imparts only a small main effect, but it may be important as a modifier of other genetic factors given the appearance of chromosome 4B in the epistasis analysis. The veracity of the 4B QTL segregating in the Carberry/AC Cadillac population is supported by the reports of QTL in the same region in durum wheat by Singh et al. (2012) and Haile et al. (2012). For example, Singh et al. (2012) reported a stem rust resistance QTL at *wPt*-*0872*, 24 cM from *wPt*-*6209* which was similarly approximately 20 cM from the 4B QTL we report.

We detected a number of less impressive main effect QTL, such as *QSr.spa*-*5A* on chromosome 5A which appeared only in the Canadian environment suggesting the gene is only effective to North American races. Njau et al. (2012) recently reported a QTL on chromosome 5A, but due to differences in markers between linkage maps it is difficult to ascertain whether their marker is the same as *QSr.spa*-*5A*. The appearance of *QSr.spa*-*5B.1* in Canadian and Kenyian field nurseries indicated that this was a broad APR factor. The QTL interval of *QSr.spa*-*5B.1* included *Xbarc142* which is coincident with the 5B QTL reported by Haile et al. (2012) for resistance to race Ug99 from a Kristal/Sebatel RIL population. *QSr.spa*-*5B.1* has further significance because it interacted epistatically with the 6D locus. The 5B chromosome also interacted with 4B, however, the 5B location of *QSr.spa*-*5B.2* was different from the main effect QTL *QSr.spa*-*5B.1*. It cannot be conclusively proven from our results if there are two separate QTL on 5B, although a previous report has suggested the possibility of two different QTL on chromosome 5B (Pumphrey 2012). Furthermore, the adult plant resistance gene *Sr56* has been reported on chromosome 5B (Bansal et al. 2008), but additional study is needed to determine the relationship of *Sr56* with the 5B QTL.

The *QSr.spa*-*7B* QTL, although infrequently expressed and explaining only a small amount of the phenotypic variation, did interact with 4B and 6D loci. *QSr.spa*-*7B* mapped to the same genomic region as *Sr17* (Crossa et al. 2007; Yu et al. 2011, 2012). *Lr34* (Sr57) was not segregating in the Carberry/AC Cadillac population; therefore, *QSr.spa*-*7D* is unique from *Lr34*. Furthermore *QSr.spa*-*7D* was detected only at the seedling stage and mapped near SSR marker *Xwmc273* which is in a different genomic region from *Lr34* as reported by Bansal et al. (2008). Further research will be required to validate *QSr.spa*-*7D* as real.

No QTL was significant in all environments which can be due to a combination of different reasons. Host response to the environment, pathogen response to environment, race structure, inoculum level, other diseases, as well as interactions of these factors, and systematic error such different raters all influence repeatability. The presence of *Lr34* in the population background likely contributed to the absence of consistently highly susceptible lines. The parents of the Carberry/AC Cadillac population are elite cultivars and were noted to possess some level of resistance to stem rust in registration testing (DePauw et al. 2011). AC Cadillac contributed most of the stem rust resistance with DH lines containing molecular variants for this parent having lower mean disease than DH lines containing corresponding Carberry molecular variants. Because both parents contributed different genetic factors, opportunity exists to harness transgressive segregation to make further improvements in stem rust resistance. However, like *Lr34*, there may be other resistance QTL, particularly to North American races, common between the parents that did not segregate and were not detected in this population.

Understanding gene or QTL interactions is necessary to know if a particular gene will function effectively in a particular background and if it will have a positive additive or epistatic effect. To dissect interactions between loci of complex traits like quantitatively inherited rust resistance, the MLM approach (Wang et al. 1999) has been used to enhance the accuracy and power for detection of QTL with epistatic effects. Several significant epistatic interactions were identified for seedling infection type, and adult plant stage severity and infection response. Both Carberry and AC Cadillac were contributors of positive molecular variants which demonstrates the importance of considering epistatic interaction in marker assisted breeding involving quantitative loci. It also points to the complexity of these interactions as seen with multiple epistatic interactions identified for stem rust infection response. The results generated from our study will provide insight into QTL × QTL interactions that can assist breeders and geneticists to develop a better understanding of genetic architecture of the complex trait, quantitative resistance to stem rust. The QTL × QTL interactions identified in this study have not been reported in previous stem rust QTL epistasis studies (Yu et al. 2011; Haile et al. 2012; Yu et al. 2012).

It is important for QTL studies to include multiple environments to determine the breadth of response of genotypes to variation in local races and to understand interactions. Although we obtained different results between Kenyan and Canadian nurseries, there were also similarities that could be valuable to study and improve resistance to Ug99 in the absence of these African races being present, for example, in Canadian disease nurseries. In other words, non-African nurseries can be used as a surrogate for further evaluating race non-specific resistance. Extensive testing leads to a greater understanding of each QTL by revealing the QTL stability across environments and the degree of phenotypic value. The use of two diverse locations and multiple years along with the measure of different facets of stem rust resistance expression through measurements of field severity, response to infection, and seedling infection type, gave us confidence in the validity of the identified genetic factors for stem rust resistance. We also investigated PBC because lines with lower PBC are generally preferred by breeders during selection. Although we determined PBC can be independent of stem rust resistance in the case of the 6A locus, the PBC-governing-regions identified on chromosome 2B and 3B will require further investigation for their role in stem rust resistance as either a linked or pleiotropic trait.

Fine mapping in the region of the QTL we identified will be necessary for their practical use in marker assisted breeding. To this end, we are studying further several hundred DH lines from the cross of Carberry/AC Cadillac. Ultimately through association with currently established sequencing projects in wheat, the QTL regions can be specifically targeted using sequence capture arrays to enrich marker depth with SNPs which can be used to identify candidate genes; the goal being to design perfect or diagnostic markers.

In summary our results indicated multiple loci influencing stem rust resistance from both Carberry and AC Cadillac with varying levels of expression under different environments. Several of the loci expressed as APR and a number of the loci were epistatic including the major partial resistance gene (*SrCad*) on 6D. Efforts to genetically dissect resistance are continually required to know how to systematically reassemble the genes in future rust resistance breeding responding to new, virulent races such as Ug99 that continue to evolve. The results demonstrate the opportunity to pyramid several genes including APR to extend resistance through transgressive segregation and presumably attain durable stem rust resistance. The identification of stem rust resistance QTL in elite lines will allow quicker application and utilization in the ongoing development of superior cultivars for farmers.

## References

[CR1] Bansal UK, Bossolini E, Miah H, Keller B, Park RF, Bariana HS (2008). Genetic mapping of seedling and adult plant stem rust resistance in two European winter wheat cultivars. Euphytica.

[CR2] Bariana HS, Hayden MJ, Ahmed NU, Bell JA, Sharp PJ, McIntosh RA (2001). Mapping of durable adult plant and seedling resistances to stripe rust and stem rust diseases in wheat. Aust J Agric Res.

[CR3] Bhavani S, Singh RP, Argillier O, Huerta-Espino J, Singh S, Njau P, Brun S, Lacam S, Desmouceaux N, McIntosh R (2011). Mapping durable adult plant stem rust resistance to the race Ug99 group in six CIMMYT wheats. Proceedings of Borlaug global rust initiative technical workshop Saint Paul.

[CR4] Crossa J, Burgueno J, Dreisigacker S, Vargas M, Herrera-Foessel SA, Lillemo M (2007). Association analysis of historical bread wheat germplasm using additive genetic covariance of relatives and population structure. Genetics.

[CR5] DePauw RM, Thomas JB, Knox RE, Clarke JM, Fernandez MR, McCaig TN, McLeod JG (1998). AC Cadillac hard red spring wheat. Can J Plant Sci.

[CR6] DePauw RM, Knox RE, McCaig TN, Clarke FR, Clarke JM (2011). Carberry hard red spring wheat. Can J Plant Sci.

[CR7] Fetch TG, McCallum BD, Menzies JG, Rashid KY, Tenuta AU (2011). Rust diseases in Canada. Prairie Soils and Crops.

[CR8] Haile JK, Nachit MM, Hammer K, Badebo A, Röder MS (2012) QTL mapping of resistance to race Ug99 of *Puccinia graminis f. sp. tritici* in durum wheat (*Triticum durum* Desf.) Mol Breeding 30:1479–1493

[CR9] Hale IL, Mamuya I, Singh D (2012) *Sr31*-virulent races (TTKSK, TTKST, and TTTSK) of the wheat stem rust pathogen *Puccinia graminis f. sp. tritici* are present in Tanzania. Plant Dis Accepted for publication 10.1094/PDIS-06-12-0604-PDN 30722237

[CR10] Hare RA, McIntosh RA (1979). Genetic and cytogenetic studies of durable adult-plant resistances in Hope and related cultivars to wheat rusts. J Plant Breeding.

[CR11] Hiebert CW, Fetch TG, Zegeye T, Thomas JB, Somers DJ, Humphreys DG, McCallum BD, Cloutier S, Singh D, Knott DR (2011). Genetics and mapping of seedling resistance to Ug99 stem rust in Canadian wheat cultivars ‘Peace’ and ‘AC Cadillac’. Theor Appl Genet.

[CR12] Hodson DP, Grønbech-Hansen J, Lassen P, Alemayehu Y, Arista J, Sonder K, Kosina P, Moncada P, Nazari K, Park RF, Pretorius ZA, Szabo LJ, Fetch T and Jin Y (2012) Tracking the wheat rust pathogens. Proceedings Borlaug Global Rust Initiative 2012 Technical Workshop, China, pp11-22

[CR13] Jin Y, Szabo LJ, Pretorius ZA, Singh RP, Ward R, Fetch T (2008). Detection of virulence to resistance gene Sr24 within race TTKS of *Puccinia graminis f. sp. tritici*. Plant Dis.

[CR14] Jin Y, Szabo LJ, Rouse MN, Fetch T, Pretorius ZA, Wanyera R, Njau P (2009). Detection of virulence to resistance gene *Sr36* within race TTKS lineage of *Puccinia graminis f.* sp*. tritici*. Plant Dis.

[CR15] Johnson R (1984). A critical analysis of durable resistance. Annu Rev Phytopathol.

[CR16] Kaur J, Bansal UK, Khana R, Saini RG, Bariana HS (2009). Molecular mapping of stem rust resistance in HD2009/WL711 recombinant inbred line population. Int J Plant Breed.

[CR17] Knox RE, Clarke JM, DePauw RM (2000). Dicamba and growth condition effects on doubled haploid production in durum wheat crossed with maize. Plant Breed.

[CR18] Kolmer JA, Garvin DF, Jin Y (2011). Expression of a thatcher wheat adult plant stem rust resistance QTL on chromosome arm 2BL is enhanced by *Lr34*. Crop Sci.

[CR19] McIntosh RA, Wellings CR, Park RF (1995). Wheat rusts, an atlas of resistance genes.

[CR20] McNeil MD, Kota R, Paul E, Dunn D, McLean R, Feuillet C, Li D, Kong X, Lagudah E, Zhang JC, Jia JZ, Spielmeyer W, Bellgard M, Appels R (2008). BAC-derived markers for assaying the stem rust resistance gene, *Sr2*, in wheat breeding programs. Mol Breeding.

[CR21] Njau P, Bhavani S, Huerta-Espino J, Keller B, Singh RP (2012). Identification of QTL associated with durable adult plant resistance to stem rust race Ug99 in wheat cultivar ‘Pavon 76′. Euphytica.

[CR22] Park R, Fetch T, Hodson D, Jin Y, Nazari K, Prashar M, Pretorius ZA (2011). International surveillance of wheat rust pathogens: progress and challenges. Euphytica.

[CR23] Peterson RF, Campbell AB, Hannah AE (1948). A diagrammatic scale for estimating rust intensity of leaves and stem of cereals. Can J Res C.

[CR24] Peturson B (1958). Wheat stem rust epidemics in western Canada in 1953, 1954, and 1955. Can J Plant Sci.

[CR25] Pretorius ZA, Singh RP, Wagoire WW, Payne TS (2000) Detection of virulence to wheat stem rust resistance gene *Sr31* in *Puccinia graminis. f.* sp. *tritici* in Uganda. Plant Dis 84:203 10.1094/PDIS.2000.84.2.203B 30841334

[CR26] Pretorius ZA, Szabo LJ, Boshoff WHP, Herselman L, Visser B (2012). First report of a new TTKSF race of wheat stem rust (*Puccinia graminis f.* sp. *tritici*) in South Africa and Zimbabwe. Plant Dis.

[CR27] Prins R, Pretorius ZA, Bender CM, Lehmensiek A (2010). QTL mapping of stripe, leaf and stem rust resistance genes in a Kariega × Avocet S doubled haploid wheat population. Mol Breed.

[CR28] Pumphrey MO (2012) Stocking the breeder’s toolbox: An update on the status of resistance to stem rust in wheat. Proceedings Borlaug Global Rust Initiative 2012 Technical Workshop, China, pp 23–29

[CR29] Singh RP, Huerta-Espino J, Rajaram S (2000). Achieving near-immunity to leaf and stripe rusts in wheat by combining slow rusting resistance genes. Acta Phytopathol Entomol Hung.

[CR30] Singh RP, Hodson DP, Huerta-Espino J, Jin Y, Bhavani S, Njau P, Herrera-Foessel S, Singh PK, Singh S, Govindan V (2011). The emergence of Ug99 races of the stem rust fungus is a threat to world wheat production. Ann Rev of Phytopathol.

[CR31] Singh A, Pandey MP, Singh AK, Knox RE, Ammar K, Clarke JM, Clarke F, Singh RP, Pozniak CJ, DePauw RM, McCallum B, Cuthbert RD, Randhawa HS, Fetch T (2012). Identification and mapping of leaf, stem and stripe rust resistance QTL and their interactions in durum wheat. Mol Breed.

[CR32] Stakman EC, Stewart DM, Loegering WQ (1962) Identification of physiologic races of *Puccinia graminis var. tritici*. USDA Agricultural Research Service E617, pp 53

[CR33] Van Ooijen JW (2006). JoinMap^®^ 4,Software for the calculation of genetic linkage maps in experimental populations.

[CR34] Van Ooijen JW (2009). MapQTL^®^ 6, Software for the mapping of quantitative trait loci in experimental populations of diploid species.

[CR35] Wang DL, Zhu J, Li ZK, Paterson AH (1999). Mapping QTLs with epistatic effects and QTL × environment interactions by mixed linear model approaches. Theor Appl Genet.

[CR36] Watson IA, de Souza CAN (1983). Long distance transport of spores of *Puccinia graminis tritici* in the southern hemisphere. Proc Linn Soc NSW.

[CR37] Yang J, Hu C, Hu H, Yu RD, Xia Z, Ye X, Zhu J (2008). QTLNetwork: mapping and visualizing genetic architecture of complex traits in experimental populations. Bioinformatics.

[CR38] Yu LX, Lorenz A, Rutkoski J, Singh RP, Bhavani S, Huerta-Espino J, Sorrells ME (2011). Association mapping and gene–gene interaction for stem rust resistance in CIMMYT spring wheat germplasm. Theor Appl Genet.

[CR39] Yu LX, Morgounov A, Wanyera R, Keser M, Singh SK, Sorrells M (2012). Identification of Ug99 stem rust resistance loci in winter wheat germplasm using genome-wide association analysis. Theor Appl Genet.

